# Classical Modeling of a Lossy Gaussian Bosonic Sampler

**DOI:** 10.3390/e26060493

**Published:** 2024-06-05

**Authors:** Mikhail V. Umanskii, Alexey N. Rubtsov

**Affiliations:** 1 Department of Physics, Lomonosov Moscow State University, Leninskie Gory 1, 119991 Moscow, Russia; mix.umanskiy@yandex.ru; 2 Russian Quantum Center, Bolshoy Bulvar 30, bld. 1, Skolkovo, 121205 Moscow, Russia

**Keywords:** Gaussian boson sampling, quantum complexity, emulation of quantum devices

## Abstract

Gaussian boson sampling (GBS) is considered a candidate problem for demonstrating quantum advantage. We propose an algorithm for the approximate classical simulation of a lossy GBS instance. The algorithm relies on the Taylor series expansion, and increasing the number of terms of the expansion that are used in the calculation yields greater accuracy. The complexity of the algorithm is polynomial in the number of modes given the number of terms is fixed. We describe conditions for the input state squeezing parameter and loss level that provide the best efficiency for this algorithm (by efficient, we mean that the Taylor series converges quickly). In recent experiments that claim to have demonstrated quantum advantage, these conditions are satisfied; thus, this algorithm can be used to classically simulate these experiments.

## 1. Introduction

Quantum computers are computational devices which operate using phenomena described by quantum mechanics. Therefore, they can carry out the operations which are not available for classical computers. The ability of a quantum computer to solve a specific task faster than any classical computer is usually referred to as quantum advantage. Although quantum algorithms that provide exponential speedup over classical ones are known, they are hard to implement in practice. Examples of such algorithms include Shor’s algorithm of factoring integers [1], that works in polynomial time, whereas all classical algorithms require exponential time. Modern quantum computers are far from experimentally demonstrating quantum advantage on basic problems like integer factorization.

Boson sampling [2] is a problem that was proposed as a good candidate for demonstrating quantum advantage due to its nature. A boson sampler is a linear-optical device that consists of non-classical sources of indistinguishable photons, a multichannel interferometer mixing photons of different sources, and photon detectors at the output channels of the interferometer. In the original proposal, the indistinguishable photons were prepared in Fock states. The problem then is to calculate the photon statistics after the interferometer given an input state and the interferometer matrix. The relevant parameters are the number of modes *N* and the total number of photons injected in the interferometer *M*. Experimentally, it corresponds to performing multiple measurements of the photon counts at the outputs of such a device [3].

Due to the technological complexity of generating Fock states, several variants of the original boson sampling problem have been proposed. They aim at improving the photon generation efficiency and increasing the scale of implementations. One such example is the scattershot boson sampling, which uses many parametric down-conversion sources to improve the single photon generation rate. It has been implemented experimentally using a 13-mode integrated photonic chip and six PDC photon sources [4].

Another variant is the Gaussian boson sampling [5,6], in which Gaussian states are injected into the interferometer instead of Fock states. Gaussian input states can be generated using PDC sources, and it allows the non-classical input states to be prepared deterministically. In this variant, the relative input photon phases can affect the sampling distribution. Experiments were carried out with *N* = 12 [7], *N* = 100 [8] and N=144 [9,10], with up to 255 photons registered in one event. The latter implementations used PPKTP crystals as PDC sources and employed an active phase-locking mechanism to ensure a coherent superposition.

Any experimental setup, of course, differs from the idealized model considered in theoretical modeling. Bosonic samplers suffer from two fundamental types of imperfections. First, the parameters of a real device, such as the reflection coefficients of the beam splitters and the phase rotations, are never known exactly. A small change in the interferometer parameters can affect the sampling statistics drastically, so that the modeling of an ideal device no longer makes much sense. Another type of imperfections is photon losses. These losses happen because of imperfections in photon preparation, absorption inside the interferometer and imperfect detectors and coupling.

There are different ways of modeling losses: for example, by introducing extra beam splitters [11] or replacing the interferometer matrix by a combination of lossless linear optics transformations and the diagonal matrix that contains transmission coefficients [12]. In the algorithm described in this paper, we will assume that losses occur on the inputs of the interferometer, and we will describe the exact way that we model them.

Imperfections in middle-sized systems make them, in general, easier to emulate with classical computers [13]. It was shown [14] that with the increase of losses in a system, the complexity of the task decreases. When the number of photons $M^{\prime }$ that arrive at the outputs is less than $\sqrt{M}$, the problem of boson sampling can be efficiently solved using classical computers. On the other hand, if the losses are low, the problem remains hard for classical computers [15].

In this paper, we propose a classical algorithm for calculating probabilities of output states in a GBS problem. The algorithm uses Taylor series expansion, and it converges faster depending on the parameters of the problem: namely, the amount of losses in the system and the squeezing parameter of the input states. The higher the losses in the system, the less orders of the series are needed to approximate the probability of observing a given output state.

The work by Oh et al. [16] used the following approach to simulating GBS: the covariance matrix of the output Gaussian state was decomposed into “quantum” and “classical” parts, in which the “quantum” part was simulated using matrix product states and the “classical” part was simulated by random displacement. Thus, when the photon loss rate is high, the computational complexity of this algorithm is reduced.

The algorithm that we propose in this paper uses some similar ideas: namely, the zeroth order of the Taylor series may be considered the “classical” part that is computed quite easily, while the remaining terms are the “quantum” part that is more computationally complex. The contribution of this “quantum” part is smaller when the losses in the system are high; thus, our algorithm also has optimal conditions that depend on the magnitude of losses. We also analyze some recent GBS implementations to compare the conditions in those experiments with the optimal conditions for our algorithm.

## 2. Problem Specification

Let us first consider a lossless linear-optics interferometer with a transmission matrix *U*:

(1) $${\hat{a}}_{i}^{†}=\sum_{j}U_{ij}{\hat{d}}_{j}^{†},\;\;\;{\hat{a}}_{i}=\sum_{j}U_{ij}^{*}{\hat{d}}_{j}$$

where creation operators acting on the *i*-th input and output modes are denoted $a_{i}^{†}$ and $d_{i}^{†}$. Suppose the input modes are injected with single-mode squeezed states:

(2) $$|\psi \rangle =e^{\sum_{i}\frac{\alpha_{i}}{2}{\left({\hat{a}}_{i}^{†}\right)}^{2}}|0\rangle ,$$

where we omit the state’s normalizing constant ${\left(1-|\alpha \right|}^{2}{)}^{N/4}$.

The goal is to calculate the probability of detecting $n_{1}$ photons in the first output mode, $n_{2}$ photons in the second output mode and so on. This probability can be calculated in the following way:

(3) $$Tr\left\{{\hat{\rho }}_{out}\hat{\vec{n}}\right\}=Tr\left\{{\hat{\rho }}_{out}\underset{i}{⨂}|n_{i}\rangle \langle n_{i}|\right\},$$

where ${\hat{\rho }}_{out}$ is the density matrix of the output state.

### Modeling Losses

In real-life bosonic samplers, there will always be losses. Here, we will model them by substituting

(4) $$a_{i}^{†}⟶ca_{i}^{†}+sb_{i}^{†},$$

where $b_{i}^{†}$ acts on a mode that we cannot observe, and $c^{2}+s^{2}=1$, c,s∈R. Now, the goal is to compute the same probability ($Tr\left\{\hat{\rho_{out}}\hat{\vec{n}}\right\}$) but taking losses into account. The input state will now be

(5) $$|\psi^{\prime }\rangle =e^{\sum_{i}\frac{\alpha_{i}}{2}{\left(c{\hat{a}}_{i}^{†}+s{\hat{b}}_{i}^{†}\right)}^{2}}|0_{a}0_{b}\rangle$$

and we now take partial trace over all loss modes when calculating the density matrix:

(6) $$\hat{\rho }=Tr_{b}\left\{e^{\sum_{i}\frac{\alpha_{i}}{2}{\left(c{\hat{a}}_{i}^{†}+s{\hat{b}}_{i}^{†}\right)}^{2}}|0_{a}0_{b}\rangle \langle 0_{a}0_{b}|e^{\sum_{i}\frac{\alpha_{i}}{2}{\left(c{\hat{a}}_{i}^{†}+s{\hat{b}}_{i}^{†}\right)}^{2}}\right\}.$$

## 3. Algorithm Derivation

Let us consider a single mode:

(7) $$|\psi^{\prime }\rangle =e^{\frac{\alpha }{2}{\left(c{\hat{a}}^{†}+s{\hat{b}}^{†}\right)}^{2}}|0_{a}0_{b}\rangle ,$$

(8) $$\hat{\rho }=Tr_{b}\left\{e^{\frac{\alpha }{2}{\left(c{\hat{a}}^{†}+s{\hat{b}}^{†}\right)}^{2}}|0_{a}0_{b}\rangle \langle 0_{a}0_{b}|e^{\frac{\alpha }{2}{\left(c\hat{a}+s\hat{b}\right)}^{2}}\right\}.$$

### 3.1. Calculating Partial Trace

We start by applying the Hubbard–Stratonovich transformation [17,18]

(9) $$e^{\frac{{\hat{A}}^{2}}{2}}=\frac{1}{\sqrt{2\pi }}\int_{-\infty }^{+\infty }e^{\xi \hat{A}-\frac{\xi^{2}}{2}}d\xi$$

to both exponents in the density matrix operator. This gives us the following:

(10) $$\begin{array}{cc} \hat{\rho } & =\frac{1}{2\pi }\int_{-\infty }^{+\infty }\int_{-\infty }^{+\infty }Tr_{b}\left\{e^{\xi \sqrt{\alpha }\left(c{\hat{a}}^{†}+s{\hat{b}}^{†}\right)}|0_{a}0_{b}\rangle \langle 0_{a}0_{b}|e^{\tilde{\xi }\sqrt{\alpha }\left(c\hat{a}+s\hat{b}\right)}\right\}e^{-\frac{\xi^{2}+{\tilde{\xi }}^{2}}{2}}d\xi d\tilde{\xi }\\ \; & =\frac{1}{2\pi \alpha }\int_{-\infty }^{+\infty }\int_{-\infty }^{+\infty }Tr_{b}\left\{e^{\xi \sqrt{\alpha }\left(c{\hat{a}}^{†}+s{\hat{b}}^{†}\right)}|0_{a}0_{b}\rangle \langle 0_{a}0_{b}|e^{\tilde{\xi }\sqrt{\alpha }\left(c\hat{a}+s\hat{b}\right)}\right\}e^{-\frac{{\left(\xi \sqrt{\alpha }\right)}^{2}+{\left(\tilde{\xi }\sqrt{\alpha }\right)}^{2}}{2\alpha }}d\left(\xi \sqrt{\alpha }\right)d\left(\tilde{\xi }\sqrt{\alpha }\right). \end{array}$$

Let us redefine $\xi \sqrt{\alpha }⟶\xi$, $\tilde{\xi }\sqrt{\alpha }⟶\tilde{\xi }$ for convenience:

(11) $$\hat{\rho }=\frac{1}{2\pi \alpha }\int_{-\infty }^{+\infty }\int_{-\infty }^{+\infty }Tr_{b}\left\{e^{\xi (c{\hat{a}}^{†}+s{\hat{b}}^{†})}|0_{a}0_{b}\rangle \langle 0_{a}0_{b}|e^{\tilde{\xi }\left(c\hat{a}+s\hat{b}\right)}\right\}e^{-\frac{\xi^{2}+{\tilde{\xi }}^{2}}{2\alpha }}d\xi d\tilde{\xi }.$$

We can now calculate the partial trace over loss modes:

(12) $$\begin{array}{cc} \; & Tr_{b}\left\{e^{\xi (c{\hat{a}}^{†}+s{\hat{b}}^{†})}|0_{a}0_{b}\rangle \langle 0_{a}0_{b}|e^{\tilde{\xi }\left(c\hat{a}+s\hat{b}\right)}\right\}\\ \; & =e^{\xi c{\hat{a}}^{†}}\left|0_{a}\rangle \langle 0_{a}\right|e^{\tilde{\xi }c\hat{a}}\cdot Tr\left\{e^{\xi s{\hat{b}}^{†}}|0_{b}\rangle \langle 0_{b}|e^{\tilde{\xi }s\hat{b}}\right\}\\ \; & =e^{\xi c{\hat{a}}^{†}}\left|0_{a}\rangle \langle 0_{a}\right|e^{\tilde{\xi }c\hat{a}}\cdot \langle 0_{b}|e^{\tilde{\xi }s\hat{b}}e^{\xi s{\hat{b}}^{†}}|0_{b}\rangle . \end{array}$$

The following expression can be simplified:

(13) $$\begin{array}{c} \langle 0_{b}|e^{\tilde{\xi }s\hat{b}}e^{\xi s{\hat{b}}^{†}}|0_{b}\rangle \\ =\langle 0_{b}|\left(1+\tilde{\xi }s\hat{b}+\frac{1}{2}{\left(\xi s\hat{b}\right)}^{2}+\ldots \right)\left(1+\xi s\hat{b^{†}}+\frac{1}{2}{\left(\xi s\hat{b^{†}}\right)}^{2}+\ldots \right)|0_{b}\rangle \\ =\left(\langle 0_{b}|+\tilde{\xi }s\langle 1_{b}|+\frac{1}{\sqrt{2}}{\left(\tilde{\xi }s\right)}^{2}\langle 2_{b}|+\ldots \right)\left(\langle 0_{b}+\xi s|1_{b}\rangle +\frac{1}{\sqrt{2}}{\left(\xi s\right)}^{2}|2_{b}\rangle +\ldots \right)\\ =1+\xi \tilde{\xi }s^{2}+\frac{1}{2}{\left(\xi \tilde{\xi }s^{2}\right)}^{2}+\ldots =e^{\xi \tilde{\xi }s^{2}}. \end{array}$$

The density matrix now can be written in the following way:

(14) $$\hat{\rho }=\frac{1}{2\pi \alpha }\int_{-\infty }^{+\infty }\int_{-\infty }^{+\infty }e^{\xi c{\hat{a}}^{†}}\left|0\rangle \langle 0\right|e^{\tilde{\xi }c\hat{a}}\cdot e^{-\frac{\xi^{2}+{\tilde{\xi }}^{2}}{2\alpha }+\xi \tilde{\xi }s^{2}}d\xi d\tilde{\xi }.$$

### 3.2. Switching between Probability Density Functions

We can view this integral as taking an expected value over a two-dimensional normal distribution. ξ and $\tilde{\xi }$ then become normally distributed random variables with a mean vector equal to zero. Their covariance matrix has the following form:

(15) $$\Sigma ={\left(\begin{array}{cc} 1/\alpha & -s^{2}\\ -s^{2} & 1/\alpha \end{array}\right)}^{-1}=\frac{1}{1/\alpha^{2}-s^{4}}\left(\begin{array}{cc} 1/\alpha & s^{2}\\ s^{2} & 1/\alpha \end{array}\right).$$

Then, we can write

(16) $$\begin{array}{cc} \hat{\rho } & =\frac{{\left(det\Sigma \right)}^{1/2}}{\alpha }\frac{1}{2\pi {\left(det\Sigma \right)}^{1/2}}\int_{-\infty }^{+\infty }\int_{-\infty }^{+\infty }e^{\xi c{\hat{a}}^{†}}\left|0\rangle \langle 0\right|e^{\tilde{\xi }c\hat{a}}e^{-\frac{\xi^{2}+{\tilde{\xi }}^{2}}{2\alpha }+\xi \tilde{\xi }s^{2}}d\xi d\tilde{\xi }\\ \; & =\frac{{\left(det\Sigma \right)}^{1/2}}{\alpha }\cdot E_{N(0,\Sigma )}\left[e^{\xi c{\hat{a}}^{†}}\left|0\rangle \langle 0\right|e^{\tilde{\xi }c\hat{a}}\right], \end{array}$$

where $E_{N(0,\Sigma )}$ denotes averaging over the two-dimensional normal distribution N(0,Σ).

The expression $e^{\xi c{\hat{a}}^{†}}\left|0\rangle \langle 0\right|e^{\tilde{\xi }c\hat{a}}$ is troublesome to calculate, since there are two different variables ξ and $\tilde{\xi }$. We want to arrive somehow at an expression with only one such variable, i.e., $e^{\xi c{\hat{a}}^{†}}\left|0\rangle \langle 0\right|e^{\xi c\hat{a}}$, which we will denote $\hat{\nu }\left(\xi c\right)$.

We now will choose normally distributed random variables $\xi_{0},\chi ,\tilde{\chi }\in R$ such that $\xi =\xi_{0}+\chi ,\;\tilde{\xi }=\xi_{0}+\tilde{\chi }$ and the distributions over $\xi ,\tilde{\xi }$ and $\xi_{0},\chi ,\tilde{\chi }$ have the same moments:

(17) $$\left\{\begin{array}{c} \bar{\xi^{2}}=\bar{{\left(\xi_{0}+\chi \right)}^{2}}=\bar{\xi_{0}^{2}}+2\bar{\xi_{0}\chi }+\bar{\chi^{2}},\\ \bar{{\tilde{\xi }}^{2}}=\bar{{\left(\xi_{0}+\tilde{\chi }\right)}^{2}}=\bar{\xi_{0}^{2}}+2\bar{\xi_{0}\tilde{\chi }}+\bar{{\tilde{\chi }}^{2}},\\ \bar{\xi \tilde{\xi }}=\bar{\left(\xi_{0}+\chi \right)\left(\xi_{0}+\tilde{\chi }\right)}=\bar{\xi_{0}^{2}}+\bar{\xi_{0}\chi }+\bar{\xi_{0}\tilde{\chi }}+\bar{\chi \tilde{\chi }}. \end{array}\right.$$

We have some freedom in choosing these variables; we will set $\bar{\xi_{0}\chi }=\bar{\xi_{0}\tilde{\chi }}=0$ so that $\xi_{0}⊥\;\;\;⊥\chi$ and $\xi_{0}⊥\;\;\;⊥\tilde{\chi }$. Then, the covariance matrix Γ of $\xi_{0},\chi ,\tilde{\chi }$ will be determined by one parameter $h=\bar{\chi \tilde{\chi }}$:

(18) $$\left\{\begin{array}{c} \bar{\xi^{2}}=\bar{\xi_{0}^{2}}+\bar{\chi^{2}},\\ \bar{{\tilde{\xi }}^{2}}=\bar{\xi_{0}^{2}}+\bar{{\tilde{\chi }}^{2}},\\ \bar{\xi \tilde{\xi }}=\bar{\xi_{0}^{2}}+h. \end{array}\right.$$

(19) $$\left\{\begin{array}{c} \bar{\xi_{0}^{2}}=\bar{\xi \tilde{\xi }}-h=\frac{s^{2}}{1/\alpha^{2}-s^{4}}-h,\\ \bar{\chi^{2}}=\bar{{\tilde{\chi }}^{2}}=\bar{\xi^{2}}-\bar{\xi \tilde{\xi }}+h=\frac{1/\alpha -s^{2}}{1/\alpha^{2}-s^{4}}+h=\frac{1}{1/\alpha +s^{2}}+h. \end{array}\right.$$

Note that $-\frac{1}{1/\alpha +s^{2}}\leq h\leq \frac{s^{2}}{1/\alpha^{2}-s^{4}}$. We will later find an optimal way to choose *h*. The density matrix in terms of the new variables $\xi_{0},\chi ,\tilde{\chi }\in N\left(0,\Gamma \right)$ is

(20) $$\hat{\rho }=\frac{{\left(det\Sigma \right)}^{1/2}}{\alpha }\cdot E_{N(0,\Gamma )}\left[e^{\left(\xi_{0}+\chi \right)c{\hat{a}}^{†}}\left|0\rangle \langle 0\right|e^{\left(\xi_{0}+\tilde{\chi }\right)c\hat{a}}\right].$$

Since $\xi_{0}⊥\;\;\;⊥\chi$ and $\xi_{0}⊥\;\;\;⊥\tilde{\chi }$, the distribution $E_{N(0,\Gamma )}$ can be split into a combination of distributions over $\xi_{0}$ and over $\chi ,\tilde{\chi }$. The covariance matrix Λ of χ and $\tilde{\chi }$ is

(21) $$\Lambda =\left(\begin{array}{cc} \bar{\chi^{2}} & \bar{\chi \tilde{\chi }}\\ \bar{\chi \tilde{\chi }} & \bar{{\tilde{\chi }}^{2}} \end{array}\right)=\left(\begin{array}{cc} \frac{1}{1/\alpha +s^{2}}+h & h\\ h & \frac{1}{1/\alpha +s^{2}}+h \end{array}\right),$$

and the distribution $E_{N(0,\Gamma )}$ can be written as

(22) $$N\left(0,\Gamma \right)=N\left(0,\bar{\xi_{0}^{2}}\right)\cdot N\left(0,\Lambda \right)$$

### 3.3. Taylor Series Expansion

We now consider the Taylor series of the expression $e^{\left(\xi_{0}+\chi \right)c{\hat{a}}^{†}}\left|0\rangle \langle 0\right|e^{\left(\xi_{0}+\tilde{\chi }\right)c\hat{a}}$, leaving only $\xi_{0}$ in the exponent:

(23) $$\begin{array}{c} e^{\left(\xi_{0}+\chi \right)c{\hat{a}}^{†}}\left|0\rangle \langle 0\right|e^{\left(\xi_{0}+\tilde{\chi }\right)c\hat{a}}=e^{\chi c{\hat{a}}^{†}}e^{\xi_{0}c{\hat{a}}^{†}}\left|0\rangle \langle 0\right|e^{\xi_{0}c\hat{a}}e^{\tilde{\chi }c\hat{a}}\\ =e^{\chi c{\hat{a}}^{†}}\hat{\nu }\left(\xi_{0}c\right)e^{\tilde{\chi }c\hat{a}}=\left(1+\chi c{\hat{a}}^{†}+\frac{{\left(\chi c{\hat{a}}^{†}\right)}^{2}}{2}+\ldots \right)\hat{\nu }\left(\xi_{0}c\right)\left(1+\tilde{\chi }c\hat{a}+\frac{{\left(\tilde{\chi }c\hat{a}\right)}^{2}}{2}+\ldots \right). \end{array}$$

Each term in the expression will be proportional to

$$c^{n+m}\chi^{n}{\tilde{\chi }}^{m}\cdot {\left({\hat{a}}^{†}\right)}^{n}\hat{\nu }\left(\xi_{0}c\right){\hat{a}}^{m},$$

and since $\xi_{0}⊥\;\;\;⊥\chi$ and $\xi_{0}⊥\;\;\;⊥\tilde{\chi }$, the integral over $\xi_{0},\chi ,\tilde{\chi }$ can be written as a product of integrals over $\xi_{0}$ and $\chi ,\tilde{\chi }$:

(24) $$E_{N(0,\Gamma )}\left[c^{n+m}\chi^{n}{\tilde{\chi }}^{m}\cdot {\left({\hat{a}}^{†}\right)}^{n}\hat{\nu }\left(\xi_{0}c\right){\hat{a}}^{m}\right]=c^{n+m}\cdot E_{N(0,\Lambda )}\left[\chi^{n}{\tilde{\chi }}^{m}\right]\cdot E_{N(0,\bar{\xi_{0}^{2}})}\left[{\left({\hat{a}}^{†}\right)}^{n}\hat{\nu }\left(\xi_{0}c\right){\hat{a}}^{m}\right].$$

The moments $E_{N(0,\Lambda )}\left[\chi^{n}{\tilde{\chi }}^{m}\right]$ can be calculated analytically using Wick’s probability theorem.

### 3.4. Choosing Γ

The idea consists of minimizing the “perturbation parameter” so that each subsequent order of the Taylor series expansion has less impact on the expression. Since higher orders of the expansion contain higher powers of $c^{2}$ and higher moments $E_{N(0,\Lambda )}\left[\chi^{n}{\tilde{\chi }}^{m}\right]$, and these moments can be calculated via second moments $\bar{\chi^{2}}=\bar{{\tilde{\chi }}^{2}}$ and $\bar{\chi \tilde{\chi }}=h$, the role of the “perturbation parameter” is played by $\varepsilon =c^{2}\cdot \max(\bar{\chi^{2}},|\bar{\chi \tilde{\chi }}\left|\right)$.

Let us consider the conditions that must be satisfied by *h*. Firstly, *h* must satisfy $-\frac{1}{1/\alpha +s^{2}}\leq h\leq \frac{s^{2}}{1/\alpha^{2}-s^{4}}$, because $\bar{\xi_{0}^{2}}\geq 0$ and $\bar{\chi^{2}}\geq 0$. Secondly, since Γ is a covariance matrix, its eigenvalues must be non-negative. The eigenvalues of Γ are $\bar{\xi_{0}^{2}}$, $\bar{\chi^{2}}-h$ and $\bar{\chi^{2}}+h$. Thus, *h* needs to satisfy

(25) $$\bar{\chi^{2}}+h\geq 0\Leftrightarrow \frac{1}{1/\alpha +s^{2}}+2h\geq 0\Leftrightarrow h\geq -\frac{1}{2}\frac{1}{1/\alpha +s^{2}}.$$

The minimum of $\max\left(\bar{\chi^{2}},\left|h\right|\right)$ is realized when $h=-\bar{\chi^{2}}=-\frac{1}{2}\frac{1}{1/\alpha +s^{2}}$.

### 3.5. Multimode Case

Let us apply the steps described above to the case of *N* modes. We start with an input state

(26) $$|\psi^{\prime (N)}\rangle =\prod_{i=1}^{N}e^{\frac{\alpha }{2}{\left(c{\hat{a}}_{i}^{†}+s{\hat{b}}_{i}^{†}\right)}^{2}}|0_{a}0_{b}\rangle .$$

We construct a density matrix and take the partial trace over loss modes:

(27) $$\hat{\rho }=Tr_{b}\left\{e^{\sum_{i}\frac{\alpha }{2}{\left(c{\hat{a}}_{i}^{†}+s{\hat{b}}_{i}^{†}\right)}^{2}}|0_{a}0_{b}\rangle \langle 0_{a}0_{b}|e^{\sum_{i}\frac{\alpha }{2}{\left(c{\hat{a}}_{i}+s{\hat{b}}_{i}\right)}^{2}}\right\}.$$

We apply the Hubbard–Stratonovich transformation 2N times, resulting in an integral over $\prod_{i=1}^{N}d\xi_{i}d{\tilde{\xi }}_{i}$:

(28) $$\begin{array}{c} \hat{\rho }=\frac{1}{{\left(2\pi \right)}^{N}}\int_{R^{2N}}Tr_{b}\left\{e^{\sum_{i}\xi_{i}\sqrt{\alpha }\left(c{\hat{a}}_{i}^{†}+s{\hat{b}}_{i}^{†}\right)}|0_{a}0_{b}\rangle \langle 0_{a}0_{b}|e^{\sum_{i}{\tilde{\xi }}_{i}\sqrt{\alpha }\left(c{\hat{a}}_{i}+s{\hat{b}}_{i}\right)}\right\}e^{-\sum_{i}\frac{\xi_{i}^{2}+{\tilde{\xi }}_{i}^{2}}{2}}\prod_{i}d\xi_{i}d{\tilde{\xi }}_{i}\\ =\frac{1}{{\left(2\pi \alpha \right)}^{N}}\int_{R^{2N}}Tr_{b}\left\{e^{\sum_{i}\xi_{i}\sqrt{\alpha }\left(c{\hat{a}}_{i}^{†}+s{\hat{b}}_{i}^{†}\right)}|0_{a}0_{b}\rangle \langle 0_{a}0_{b}|e^{\sum_{i}{\tilde{\xi }}_{i}\sqrt{\alpha }\left(c{\hat{a}}_{i}+s{\hat{b}}_{i}\right)}\right\}\\ \cdot e^{-\sum_{i}\frac{{\left(\xi_{i}\sqrt{\alpha }\right)}^{2}+{\left({\tilde{\xi }}_{i}\sqrt{\alpha }\right)}^{2}}{2\alpha }}\prod_{i}d\left(\xi_{i}\sqrt{\alpha }\right)d\left({\tilde{\xi }}_{i}\sqrt{\alpha }\right), \end{array}$$

where the integral for each variable $\xi_{i}$ and ${\tilde{\xi }}_{i}$ is calculated over (−∞,+∞).

Again, we redefine $\xi_{i}\sqrt{\alpha }⟶\xi_{i}$, ${\tilde{\xi }}_{i}\sqrt{\alpha }⟶{\tilde{\xi }}_{i}$:

(29) $$\hat{\rho }=\frac{1}{{\left(2\pi \alpha \right)}^{N}}\int_{R^{2N}}Tr_{b}\left\{e^{\sum_{i}\xi_{i}\left(c{\hat{a}}_{i}^{†}+s{\hat{b}}_{i}^{†}\right)}|0_{a}0_{b}\rangle \langle 0_{a}0_{b}|e^{\sum_{i}{\tilde{\xi }}_{i}\left(c{\hat{a}}_{i}+s{\hat{b}}_{i}\right)}\right\}e^{-\sum_{i}\frac{\xi_{i}^{2}+{\tilde{\xi }}_{i}^{2}}{2\alpha_{i}}}\prod_{i}d\xi_{i}d{\tilde{\xi }}_{i}.$$

We compute partial trace over loss modes:

(30) $$\hat{\rho }=\frac{1}{{\left(2\pi \alpha \right)}^{N}}\int_{R^{2N}}e^{\sum_{i}\xi_{i}c{\hat{a}}_{i}^{†}}\left|0\rangle \langle 0\right|e^{\sum_{i}{\tilde{\xi }}_{i}c{\hat{a}}_{i}}e^{-\sum_{i}\frac{\xi_{i}^{2}+{\tilde{\xi }}_{i}^{2}}{2\alpha }+\xi_{i}{\tilde{\xi }}_{i}s^{2}}\prod_{i}d\xi_{i}d{\tilde{\xi }}_{i}.$$

This expression now can be considered as taking an expected value over a 2N-dimensional normal distribution where all variable pairs $\xi_{i},{\tilde{\xi }}_{i}$ are independent. Every variable pair $\xi_{i},{\tilde{\xi }}_{i}$ has covariance matrix Σ, and we can write this expression in the following way:

(31) $$\hat{\rho }=\frac{{\left(det\Sigma \right)}^{N/2}}{\alpha^{N}}\cdot E_{\prod_{i}N\left(0,\Sigma \right)}\left[e^{\sum_{i}\xi_{i}c{\hat{a}}_{i}^{†}}\left|0\rangle \langle 0\right|e^{\sum_{i}\tilde{\xi_{i}}c{\hat{a}}_{i}}\right].$$

For each variable pair $\xi_{i},{\tilde{\xi }}_{i}$ we now choose $\xi_{0i},\chi_{i},{\tilde{\chi }}_{i}$ in a way that is described above. Then,

(32) $$\hat{\rho }=\frac{{\left(det\Sigma \right)}^{N/2}}{\alpha^{N}}\cdot E_{\prod_{i}N\left(0,\Gamma \right)}\left[e^{\sum_{i}\left(\xi_{0i}+\chi_{i}\right)c{\hat{a}}_{i}^{†}}\left|0\rangle \langle 0\right|e^{\sum_{i}\left(\xi_{0i}+{\tilde{\chi }}_{i}\right)c{\hat{a}}_{i}}\right].$$

We now consider the Taylor series expansion (up to the second order) of the expression in the square brackets, which we will denote $\hat{\mu }$:

(33) $$\begin{array}{c} \hat{\mu }=e^{\sum_{i}\chi_{i}c{\hat{a}}_{i}^{†}}e^{\sum_{i}\xi_{0i}c{\hat{a}}_{i}^{†}}\left|0\rangle \langle 0\right|e^{\sum_{i}\xi_{0i}c{\hat{a}}_{i}}e^{\sum_{i}\tilde{\chi_{i}}c{\hat{a}}_{i}}\\ =\prod_{i}\left(1+\chi_{i}c{\hat{a}}_{i}^{†}+\frac{{\left(\chi_{i}c{\hat{a}}_{i}^{†}\right)}^{2}}{2}\right)e^{\sum_{i}\xi_{0i}c{\hat{a}}_{i}^{†}}\left|0\rangle \langle 0\right|e^{\sum_{i}\xi_{0i}c{\hat{a}}_{i}}\prod_{i}\left(1+\tilde{\chi_{i}}c{\hat{a}}_{i}+\frac{{\left(\tilde{\chi_{i}}c{\hat{a}}_{i}\right)}^{2}}{2}\right). \end{array}$$

The creation operators ${\hat{a}}_{i}^{†}$ that act on the input modes can be written in terms of the operators ${\hat{d}}_{i}^{†}$ that act on the output modes:

(34) $$\begin{array}{c} \hat{\mu }=\prod_{i}\left(1+\chi_{i}c\sum_{j}U_{ij}{\hat{d}}_{j}^{†}+\frac{{\left(\chi_{i}c\sum_{j}U_{ij}{\hat{d}}_{j}^{†}\right)}^{2}}{2}\right)e^{\sum_{ij}\xi_{0i}cU_{ij}{\hat{d}}_{j}^{†}}|0\rangle \\ \cdot \langle 0|e^{\sum_{ij}\xi_{0i}cU_{ij}^{*}{\hat{d}}_{j}}\prod_{i}\left(1+\tilde{\chi_{i}}c\sum_{j}U_{ij}^{*}{\hat{d}}_{j}+\frac{{\left(\tilde{\chi_{i}}c\sum_{j}U_{ij}^{*}{\hat{d}}_{j}\right)}^{2}}{2}\right). \end{array}$$

We will denote

(35) $$\hat{\nu }\left({\vec{\xi }}_{0}c\right)=e^{\sum_{ij}\xi_{0i}cU_{ij}{\hat{d}}_{j}^{†}}\left|0\rangle \langle 0\right|e^{\sum_{ij}\xi_{0i}cU_{ij}^{*}{\hat{d}}_{j}}.$$

We can expand the brackets in the expression for $\hat{\mu }$, leaving the terms up to the second order:

(36) $$\begin{array}{c} \prod_{i}\left(1+\chi_{i}c\sum_{j}U_{ij}{\hat{d}}_{j}^{†}+\frac{{\left(\chi_{i}c\sum_{j}U_{ij}{\hat{d}}_{j}^{†}\right)}^{2}}{2}\right)=1+\sum_{j}{\hat{d}}_{j}^{†}\sum_{i}c\chi_{i}U_{ij}\\ +\sum_{jk}{\hat{d}}_{j}^{†}{\hat{d}}_{k}^{†}\left(\frac{1}{2}\sum_{i}c^{2}\chi_{i}^{2}U_{ij}U_{ik}+\sum_{i\neq l}c\chi_{i}c_{l}\chi_{l}U_{ij}U_{lk}\right). \end{array}$$

(37) $$\begin{array}{c} \prod_{i}\left(1+\tilde{\chi_{i}}c\sum_{j}U_{ij}^{*}{\hat{d}}_{j}+\frac{{\left(\tilde{\chi_{i}}c\sum_{j}U_{ij}^{*}{\hat{d}}_{j}\right)}^{2}}{2}\right)=1+\sum_{j}{\hat{d}}_{j}\sum_{i}c\tilde{\chi_{i}}U_{ij}^{*}\\ +\sum_{jk}{\hat{d}}_{j}\hat{d_{k}}\left(\frac{1}{2}\sum_{i}c^{2}{\tilde{\chi }}_{i}^{2}U_{ij}^{*}U_{ik}^{*}+\sum_{i\neq l}c\tilde{\chi_{i}}c_{l}\tilde{\chi_{l}}U_{ij}^{*}U_{lk}^{*}\right). \end{array}$$

When we take the product of these two expressions, most of the resulting terms will have zero expected value because of the properties of the normal distribution. Then

(38) $$\begin{array}{cc} \hat{\mu }= & \hat{\nu }\left({\vec{\xi }}_{0}c\right)+\frac{1}{2}\sum_{i}\chi_{i}^{2}c^{2}\sum_{jk}U_{ij}U_{ik}\cdot {\hat{d}}_{j}^{†}{\hat{d}}_{k}^{†}\hat{\nu }\left({\vec{\xi }}_{0}c\right)+\frac{1}{2}\sum_{i}{\tilde{\chi_{i}}}^{2}c^{2}\sum_{jk}U_{ij}^{*}U_{ik}^{*}\cdot \hat{\nu }\left({\vec{\xi }}_{0}c\right){\hat{d}}_{j}{\hat{d}}_{k}\\ \; & +\sum_{i}\chi_{i}\tilde{\chi_{i}}c^{2}\sum_{jk}U_{ij}U_{ik}^{*}\cdot {\hat{d}}_{j}^{†}\hat{\nu }\left({\vec{\xi }}_{0}c\right){\hat{d}}_{k}\\ \; & +\frac{1}{4}\sum_{ij}\chi_{i}^{2}{\tilde{\chi_{j}}}^{2}c^{4}\sum_{klmn}U_{ik}U_{il}U_{jm}^{*}U_{jn}^{*}\cdot {\hat{d}}_{k}^{†}{\hat{d}}_{l}^{†}\hat{\nu }\left({\vec{\xi }}_{0}c\right){\hat{d}}_{m}{\hat{d}}_{n}\\ \; & +\sum_{i\neq j}\chi_{i}\chi_{j}\tilde{\chi_{i}}\tilde{\chi_{j}}c^{4}\sum_{klmn}U_{ik}U_{jl}U_{im}^{*}U_{jn}^{*}\cdot {\hat{d}}_{k}^{†}{\hat{d}}_{l}^{†}\hat{\nu }\left({\vec{\xi }}_{0}c\right){\hat{d}}_{m}{\hat{d}}_{n}. \end{array}$$

The integrals over $\chi_{i}$, ${\tilde{\chi }}_{i}$ result in specific moments of the distribution, and the integral over $\xi_{0i}$ can be calculated using Monte-Carlo methods. The final expression is

(39) $$\begin{array}{cc} Tr\left\{{\hat{\rho }}_{out}\hat{\vec{n}}\right\} & =\frac{{\left(\det\Sigma \right)}^{N/2}}{\alpha^{N}}E_{\prod_{i}N\left(0,\bar{\xi_{0}^{2}}\right)}[Tr\left\{\hat{\nu }\left({\vec{\xi }}_{0}c\right)\hat{\vec{n}}\right\}\\ \; & +\frac{1}{2}\bar{\chi^{2}}c^{2}\sum_{ijk}U_{ij}U_{ik}\cdot Tr\left\{{\hat{d}}_{j}^{†}{\hat{d}}_{k}^{†}\hat{\nu }\left({\vec{\xi }}_{0}c\right)\hat{\vec{n}}\right\}+\frac{1}{2}\bar{{\tilde{\chi }}^{2}}c^{2}\sum_{ijk}U_{ij}^{*}U_{ik}^{*}\cdot Tr\left\{\hat{\nu }\left({\vec{\xi }}_{0}c\right){\hat{d}}_{j}{\hat{d}}_{k}\hat{\vec{n}}\right\}\\ \; & +\bar{\chi \tilde{\chi }}c^{2}\sum_{ijk}U_{ij}U_{ik}^{*}\cdot Tr\left\{{\hat{d}}_{j}^{†}\hat{\nu }\left({\vec{\xi }}_{0}c\right){\hat{d}}_{k}\hat{\vec{n}}\right\}\\ \; & +\frac{1}{4}c^{4}\sum_{ij}\left({\left(\bar{\chi^{2}}\right)}^{2}+2\delta_{ij}{\left(\bar{\chi \tilde{\chi }}\right)}^{2}\right)\sum_{klmn}U_{ik}U_{il}U_{jm}^{*}U_{jn}^{*}\cdot Tr\left\{{\hat{d}}_{k}^{†}{\hat{d}}_{l}^{†}\hat{\nu }\left({\vec{\xi }}_{0}c\right){\hat{d}}_{m}{\hat{d}}_{n}\hat{\vec{n}}\right\}\\ \; & +{\left(\bar{\chi \tilde{\chi }}\right)}^{2}c^{4}\sum_{i\neq j}\sum_{klmn}U_{ik}U_{jl}U_{im}^{*}U_{jn}^{*}\cdot Tr\left\{{\hat{d}}_{k}^{†}{\hat{d}}_{l}^{†}\hat{\nu }\left({\vec{\xi }}_{0}c\right){\hat{d}}_{m}{\hat{d}}_{n}\hat{\vec{n}}\right\}], \end{array}$$

where by Wick’s probability theorem $\bar{\chi_{i}^{2}{\tilde{\chi }}_{j}^{2}}=\bar{\chi_{i}^{2}}\cdot \bar{{\tilde{\chi }}_{j}^{2}}+\bar{\chi_{i}{\tilde{\chi }}_{j}}\cdot \bar{\chi_{i}{\tilde{\chi }}_{j}}+\bar{\chi_{i}{\tilde{\chi }}_{j}}\cdot \bar{\chi_{i}{\tilde{\chi }}_{j}}={\left(\bar{\chi^{2}}\right)}^{2}+2\delta_{ij}{\left(\bar{\chi \tilde{\chi }}\right)}^{2}$.

### 3.6. Calculating Traces

In order to calculate $Tr\left\{{\hat{\rho }}_{out}\hat{\vec{n}}\right\}$, we need to be able to calculate expressions $Tr\left\{\hat{\nu }\left(\vec{x}\right)\hat{\vec{n}}\right\}$, $Tr\left\{{\hat{d}}_{j}^{†}{\hat{d}}_{k}^{†}\hat{\nu }\left(\vec{x}\right)\hat{\vec{n}}\right\}$, $Tr\left\{\hat{\nu }\left(\vec{x}\right){\hat{d}}_{j}{\hat{d}}_{k}\hat{\vec{n}}\right\}$, $Tr\left\{{\hat{d}}_{j}^{†}\hat{\nu }\left(\vec{x}\right){\hat{d}}_{k}\hat{\vec{n}}\right\}$, etc., for different $\vec{x}$. The first one can be calculated fairly easily:

(40) $$\begin{array}{cc} Tr\left\{\hat{\nu }\left(\vec{x}\right)\hat{\vec{n}}\right\} & =Tr\left\{e^{\sum_{ij}x_{i}U_{ij}{\hat{d}}_{j}^{†}}\left|0\rangle \langle 0\right|e^{\sum_{ij}x_{i}U_{ij}^{*}{\hat{d}}_{j}}|\vec{n}\rangle \langle \vec{n}|\right\}\\ \; & =\langle 0|e^{\sum_{ij}x_{i}U_{ij}^{*}{\hat{d}}_{j}}|\vec{n}\rangle \langle \vec{n}|e^{\sum_{ij}x_{i}cU_{ij}{\hat{d}}_{j}^{†}}|0\rangle \\ \; & =\prod_{j}0e^{\sum_{i}x_{i}U_{ij}^{*}{\hat{d}}_{j}}|n_{j}\rangle \langle n_{j}|e^{\sum_{i}x_{i}U_{ij}{\hat{d}}_{j}^{†}}|0\rangle \\ \; & =\prod_{j}\langle 0|\frac{{\left(\sum_{i}x_{i}U_{ij}^{*}{\hat{d}}_{j}\right)}^{n_{j}}}{n_{j}!}|n_{j}\rangle \langle n_{j}|\frac{{\left(\sum_{i}x_{i}U_{ij}{\hat{d}}_{j}^{†}\right)}^{n_{j}}}{n_{j}!}|0\rangle \\ \; & =\prod_{j}\left[\frac{{\left(\sum_{i}x_{i}U_{ij}^{*}\right)}^{n_{j}}}{\sqrt{n_{j}!}}\right]\cdot \left[\frac{{\left(\sum_{i}x_{i}U_{ij}\right)}^{n_{j}}}{\sqrt{n_{j}!}}\right]\\ \; & =\prod_{j}\frac{1}{n_{j}!}{\left|\sum_{i}x_{i}U_{ij}\right|}^{2n_{j}}. \end{array}$$

Now, suppose we need to calculate $Tr\left\{{\left({\hat{d}}_{1}^{†}\right)}^{q_{1}}\ldots {\left({\hat{d}}_{N}^{†}\right)}^{q_{N}}\hat{\nu }\left(\vec{x}\right){\left({\hat{d}}_{1}\right)}^{q_{1}}\ldots {\left({\hat{d}}_{N}\right)}^{q_{N}}\hat{\vec{n}}\right\}$. First, we note that

(41) $$\begin{array}{c} Tr\left\{{\left({\hat{d}}_{1}^{†}\right)}^{q_{1}}\ldots {\left({\hat{d}}_{N}^{†}\right)}^{q_{N}}\hat{\nu }\left(\vec{x}\right){\left({\hat{d}}_{1}\right)}^{p_{1}}\ldots {\left({\hat{d}}_{N}\right)}^{p_{N}}\hat{\vec{n}}\right\}=Tr\left\{\hat{\nu }\left(\vec{x}\right){\left({\hat{d}}_{1}\right)}^{p_{1}}\ldots {\left({\hat{d}}_{N}\right)}^{p_{N}}\hat{\vec{n}}{\left({\hat{d}}_{1}^{†}\right)}^{q_{1}}\ldots {\left({\hat{d}}_{N}^{†}\right)}^{q_{N}}\right\}\\ =Tr\left\{\hat{\nu }\left(\vec{x}\right)|\vec{n}-\vec{p}\rangle \langle \vec{n}-\vec{q}|\right\}\cdot \prod_{j}\sqrt{n_{j}\left(n_{j}-1\right)\ldots \left(n_{j}-p_{j}+1\right)n_{j}\left(n_{j}-1\right)\ldots \left(n_{j}-q_{j}+1\right)}\\ =Tr\left\{\hat{\nu }\left(\vec{x}\right)|\vec{n}-\vec{p}\rangle \langle \vec{n}-\vec{q}|\right\}\cdot \prod_{j}\sqrt{\frac{n_{j}!}{(n_{j}-p_{j})!}\frac{n_{j}!}{(n_{j}-q_{j})!}}, \end{array}$$

where by, e.g., $|\vec{n}-\vec{p}\rangle$ we mean ${⨂}_{i}|n_{i}-p_{i}\rangle$.

(42) $$\begin{array}{cc} Tr\left\{\hat{\nu }\left(\vec{x}\right)|\vec{n}-\vec{p}\rangle \langle \vec{n}-\vec{q}|\right\} & =\prod_{j}0e^{\sum_{i}x_{i}U_{ij}^{*}{\hat{d}}_{j}}|n_{j}-p_{j}\rangle \langle n_{j}-q_{j}|e^{\sum_{i}x_{i}U_{ij}{\hat{d}}_{j}^{†}}0\\ \; & =\prod_{j}\left[\frac{{\left(\sum_{i}x_{i}U_{ij}^{*}\right)}^{n_{j}-p_{j}}}{\sqrt{(n_{j}-p_{j})!}}\right]\cdot \left[\frac{{\left(\sum_{i}x_{i}U_{ij}\right)}^{n_{j}-q_{j}}}{\sqrt{(n_{j}-q_{j})!}}\right]\\ \; & =\prod_{j}\frac{1}{\sqrt{\left(n_{j}-p_{j}\right)!\left(n_{j}-q_{j}\right)!}}\cdot \frac{{\left|\sum_{i}x_{i}U_{ij}\right|}^{2n_{j}}}{{\left(\sum_{i}x_{i}U_{ij}^{*}\right)}^{p_{j}}{\left(\sum_{i}x_{i}U_{ij}\right)}^{q_{j}}}\\ \; & =\prod_{j}\frac{{\left|\sum_{i}x_{i}U_{ij}\right|}^{2n_{j}}}{{\left(\sum_{i}x_{i}U_{ij}^{*}\right)}^{p_{j}}{\left(\sum_{i}x_{i}U_{ij}\right)}^{q_{j}}}\cdot \frac{\sqrt{\frac{n_{j}!}{(n_{j}-p_{j})!}\frac{n_{j}!}{(n_{j}-q_{j})!}}}{n_{j}!}\\ \; & =Tr\left\{\hat{\nu }\left(\vec{x}\right)\hat{\vec{n}}\right\}\prod_{j}\sqrt{\frac{n_{j}!}{(n_{j}-p_{j})!}\frac{n_{j}!}{(n_{j}-q_{j})!}}\frac{1}{{\left(\sum_{i}x_{i}U_{ij}^{*}\right)}^{p_{j}}{\left(\sum_{i}x_{i}U_{ij}\right)}^{q_{j}}}. \end{array}$$

Finally, we can write

(43) $$\begin{array}{c} Tr\left\{{\left({\hat{d}}_{1}^{†}\right)}^{q_{1}}\ldots {\left({\hat{d}}_{N}^{†}\right)}^{q_{N}}\hat{\nu }\left(\vec{x}\right){\left({\hat{d}}_{1}\right)}^{p_{1}}\ldots {\left({\hat{d}}_{N}\right)}^{p_{N}}\hat{\vec{n}}\right\}\\ =Tr\left\{\hat{\nu }\left(\vec{x}\right)\hat{\vec{n}}\right\}\prod_{j}\frac{n_{j}!}{(n_{j}-p_{j})!}\frac{n_{j}!}{(n_{j}-q_{j})!}\frac{1}{{\left(\sum_{i}x_{i}U_{ij}^{*}\right)}^{p_{j}}{\left(\sum_{i}x_{i}U_{ij}\right)}^{q_{j}}}. \end{array}$$

## 4. Algorithm Overview

The goal of the algorithm is to calculate the probability of a state $|\vec{n}\rangle$, given $\vec{n}$, α, *c*, *s* and *U*. We assume that the Taylor series expansion is calculated up to the desired order before computation starts. The integrals over $\chi_{i}$ and ${\tilde{\chi }}_{i}$ should also be computed (it can be completed analytically via Wick’s probability theorem).

We start by calculating two-variable covariance matrix Σ using α and *s*. We now select Γ in the way specified above such that it minimizes the series expansion parameter ε. In order to compute the integrals over $\xi_{0i}$, we sample $\xi_{0i}$ for each *i* from a normal distribution $N(0,\bar{\xi_{0}^{2}})$.

We now compute $Tr\left\{\hat{\mu }\hat{\vec{n}}\right\}$, which by linearity consists in computing traces of the form described above; for each sample, ${\vec{\xi }}_{0}$ we need only a polynomial number of operations.

Finally, we take an average over our samples and multiply by the necessary constant ${\left(\frac{\sqrt{\det\Sigma }}{\alpha \sqrt{\left(1-|\alpha {|}^{2}\right)}}\right)}^{N}$.

## 5. Taylor Series Convergence for Actual Experimental Conditions

We have discussed above the fact that the role of the “perturbation parameter” in the series expansion is played by $c^{2}\cdot \max\left(\bar{\chi^{2}},\left|h\right|\right)$, which we can choose to be equal to $\varepsilon =\frac{1}{2}\frac{c^{2}}{1/\alpha +s^{2}}$. This parameter depends on the experimental conditions (i.e., the squeezing parameter of the input state α and loss level $s^{2}$). The smaller this parameter is, the faster the series will converge. Thus, the best conditions for this algorithm are achieved when the loss level $s^{2}$ is high and the squeezing parameter α is low. Let us consider the actual experimental implementation of the Gaussian boson sampling problem and estimate how small this parameter is in those conditions.

Let us consider the relation between α and the average amount of photons per state 〈n〉. If the squeezing parameter is $\zeta =re^{i\varphi }$, then α=tanhr, while $\langle n\rangle =\sinh^{2}r$.

In a paper by Zhong et al. [8], 25 PPKTP crystals were used to produce 25 two-mode squeezed states, which is equivalent to 50 single-mode squeezed states. The average number of photons registered by the detectors was 43. Thus, the average amount of photons per mode 〈n〉 is around $\frac{43}{50}$; $r=\mathrm{arcsinh}(\sqrt{\langle n\rangle })\approx 0.855$, α=tanhr≈0.694. The average collection efficiency is said to be $c^{2}=0.628$. Then, $\varepsilon =\frac{1}{2}\frac{c^{2}}{\frac{1}{\alpha }+s^{2}}\approx 0.18$.

In another paper by Zhong et al. [9], the average amount of photons produced was increased to 70 at maximum pump intensity. This corresponds to α≈0.76. The overall transmission rate in the experiment is said in the paper to be 48% and 54% for different settings, so we take $s^{2}\approx 0.5$. This yields ε≈0.14.

In the most recent experiment by Deng et al. [10], the average amount of photons was increased even more, measuring states with ≈50, ≈75 and ≈100 photons on average with different pump intensities while still producing 25 two-mode squeezed states. The efficiency of the setup is said to be 43%, yielding ε≈0.11, ε≈0.12 and ε≈0.12, respectively.

To estimate the expected accuracy of the algorithm, we can assume that the numerical values of each order are approximately equal, meaning that we can write

(44) $$Tr\left\{{\hat{\rho }}_{out}\hat{\vec{n}}\right\}=P_{0}+\varepsilon P_{2}+\varepsilon^{2}P_{4}+\ldots ,$$

where $P_{k}$ denotes the sum of all the terms of the *k*-th order, and $P_{0}\approx P_{2}\approx P_{4}\approx P_{k}$ is assumed. The expression then becomes a geometric progression with common ratio ε. Then, on average, the 0-th order contributes to the probability a part equal to 1−ε, while the second order contributes ε(1−ε), the fourth contributes $\varepsilon^{2}\left(1-\varepsilon \right)$, etc.

Calculating up to the second order then discards a total contribution of ε, which is approximately 0.18^2^ = 3.2%, 0.14^2^ = 1.96%, 0.11^2^ = 1.21% and 0.12^2^ = 1.44% for the conditions that are analyzed above. When the calculation is performed up to the fourth order, the lost contribution is approximately 0.18^3^ ≈ 0.58%, 0.14^3^ ≈ 0.27%, 0.11^3^ ≈ 0.13% and 0.12^3^ ≈ 0.17%.

The conclusion that we draw is that even in large GBS experiments which are said to demonstrate quantum advantage, the conditions are such that ε is fairly small, and calculating up to the fourth order is enough for the lost contribution to be below 1%.

## 6. Implementation Details

### 6.1. Contraction Precomputation

Let us consider the term

$$\frac{1}{2}\bar{\chi^{2}}c^{2}\sum_{ijk}U_{ij}U_{ik}\cdot Tr\left\{{\hat{d}}_{j}^{†}{\hat{d}}_{k}^{†}\hat{\nu }\left({\vec{\xi }}_{0}c\right)\hat{\vec{n}}\right\}.$$

We can rewrite it as

(45) $$\frac{1}{2}\bar{\chi^{2}}c^{2}\sum_{jk}Tr\left\{{\hat{d}}_{j}^{†}{\hat{d}}_{k}^{†}\hat{\nu }\left({\vec{\xi }}_{0}c\right)\hat{\vec{n}}\right\}\sum_{i}U_{ij}U_{ik}=\frac{1}{2}\bar{\chi^{2}}c^{2}\sum_{jk}Tr\left\{{\hat{d}}_{j}^{†}{\hat{d}}_{k}^{†}\hat{\nu }\left({\vec{\xi }}_{0}c\right)\hat{\vec{n}}\right\}T_{jk},$$

where $T_{jk}=\sum_{i}U_{ij}U_{ik}$ is a contraction of *U* with itself. It depends only on *U* and can be calculated before sampling ${\vec{\xi }}_{0}$, which reduces the amount of operations required to calculate each probability sample from a ${\vec{\xi }}_{0}$ sample.

### 6.2. Factorial Fractions Precomputation

In calculating traces of the form described above, we need to calculate factorial fractions of the form $\frac{m!}{(m-p)!}\equiv F_{p}^{m}$, where 0≤p≤m. Since the target state $\hat{\vec{n}}$ is fixed, $m\leq \max(n_{i})$.

### 6.3. Reusing $\sum_{i}x_{i}U_{ij}$

During calculation, while calculating each trace, we can calculate $\sum_{i}x_{i}U_{ij}$ only once for each ${\vec{\xi }}_{0}$ sample and then reuse it, thus using less operations to calculate each trace. Let us denote $S_{j}=\sum_{i}x_{i}U_{ij}$; $\vec{S}=U^{T}\vec{x}$. Then,

(46) $$Tr\left\{\hat{\nu }\left(\vec{x}\right)\hat{\vec{n}}\right\}=\prod_{j}\frac{1}{n_{j}!}{\left|S_{j}\right|}^{2n_{j}}$$

and

(47) $$\begin{array}{c} Tr\left\{{\left({\hat{d}}_{1}^{†}\right)}^{q_{1}}\ldots {\left({\hat{d}}_{N}^{†}\right)}^{q_{N}}\hat{\nu }\left(\vec{x}\right){\left({\hat{d}}_{1}\right)}^{p_{1}}\ldots {\left({\hat{d}}_{N}\right)}^{p_{N}}\hat{\vec{n}}\right\}\\ =Tr\left\{\hat{\nu }\left(\vec{x}\right)\hat{\vec{n}}\right\}\prod_{j}\frac{F_{p_{j}}^{n_{j}}F_{q_{j}}^{n_{j}}}{{\left(S_{j}^{*}\right)}^{p_{j}}{\left(S_{j}\right)}^{q_{j}}}. \end{array}$$

## 7. Complexity Analysis

### 7.1. Precomputation

In this section, we will analyze the computational complexity of precomputation. By precomputation we mean the calculations that need to be carried out only once before ${\vec{\xi }}_{0}$ sampling and before calculating probability samples for each ${\vec{\xi }}_{0}$. The multiplicative constant ${\left(\frac{\sqrt{\det\Sigma }}{\alpha \sqrt{\left(1-|\alpha {|}^{2}\right)}}\right)}^{N}$ can be calculated with O(N) multiplication operations. For each term in the resulting sum, we will define its order to be the number of variables χ and $\tilde{\chi }$, or, equivalently, the power of the loss parameter *c*. Thus, the term

$$\bar{\chi \tilde{\chi }}c^{2}\sum_{ijk}U_{ij}U_{ik}^{*}\cdot Tr\left\{{\hat{d}}_{j}^{†}\hat{\nu }\left({\vec{\xi }}_{0}c\right){\hat{d}}_{k}\hat{\vec{n}}\right\}$$

will be of the second order. Then, each term of the order *K* will have a contraction of the form

$$\sum_{j_{1}\ldots j_{K}}U_{i_{1}j_{1}}U_{i_{2}j_{2}}\ldots U_{i_{K}j_{K}}$$

where some of the $U_{ji}$ can be conjugated. This leaves at most K+1 different ways to conjugate the factors. Each contraction has *K* free indices, and calculating the sum requires $N^{K}$ additions and $N^{K}\left(K-1\right)$ multiplications. The total number of additions is $N^{2K}$ and the number of multiplications is $N^{2K}\left(K-1\right)$, where *K* is the maximum order we choose to calculate.

Calculating all $F_{p}^{m}\equiv \frac{m!}{(m-p)!}$ for $0\leq p\leq m\leq \max(n_{i})$ requires only around $\frac{\max{\left(n_{i}\right)}^{2}}{2}$ multiplications, since $\forall m\;\;F_{0}^{m}=1$, $F_{1}^{m}=m$, $F_{2}^{m}=m\left(m-1\right)=\left(m-1\right)F_{1}^{m}$, …, $F_{k}^{m}=\left(m-k+1\right)F_{k-1}^{m}$.

### 7.2. Probability Sample Computation

Here, we will analyze the computational complexity of calculating a single probability sample given ${\vec{\xi }}_{0}$. We will assume that the terms are calculated up to some order *K*.

Calculating the trace

(48) $$Tr\left\{\hat{\nu }\left(\vec{x}\right)\hat{\vec{n}}\right\}=\prod_{j}\frac{1}{n_{j}!}{\left|\sum_{i}x_{i}U_{ij}\right|}^{2n_{j}}.$$

requires one multiplication of an N×N matrix by a *N*-dimensional vector, *N* exponentiation operations and 2N multiplication operations. This calculations needs to be made only once for each $\vec{x}$. Calculating any other trace of the form

$$Tr\left\{{\left({\hat{d}}_{1}^{†}\right)}^{q_{1}}\ldots {\left({\hat{d}}_{N}^{†}\right)}^{q_{N}}\hat{\nu }\left(\vec{x}\right){\left({\hat{d}}_{1}\right)}^{p_{1}}\ldots {\left({\hat{d}}_{N}\right)}^{p_{N}}\hat{\vec{n}}\right\}$$

requires 2N exponentiation operations and 4N multiplication operations (since factorial fractions are precomputed).

The number of terms for a given order *K* is $N^{K}$ times the number of different non-zero *K*-th order moments $\bar{\chi_{i_{1}}\cdots \chi_{i_{r}}{\tilde{\chi }}_{i_{r+1}}\cdots {\tilde{\chi }}_{i_{K}}}$. The exact amount is hard to calculate, but the total number of moments (including those that are zero) is $\left(K+1\right)N^{K}$. Thus, the maximum amount of terms required to compute is $\left(K+1\right)N^{2K}$.

Since the amount of operations required to calculate each term is O(N), the total computational complexity of calculating a probability sample for a given ${\vec{\xi }}_{0}$ is $O\left(K\cdot N^{2K}\right)$.

## 8. Results

Below are the results of probability calculation for N=5 for different output states. The calculated probabilities are compared to exact solutions. The parameters are α=0.9, $c=s=\frac{\sqrt{2}}{2}$. The number of samples is 4096.

**Figure 1:**
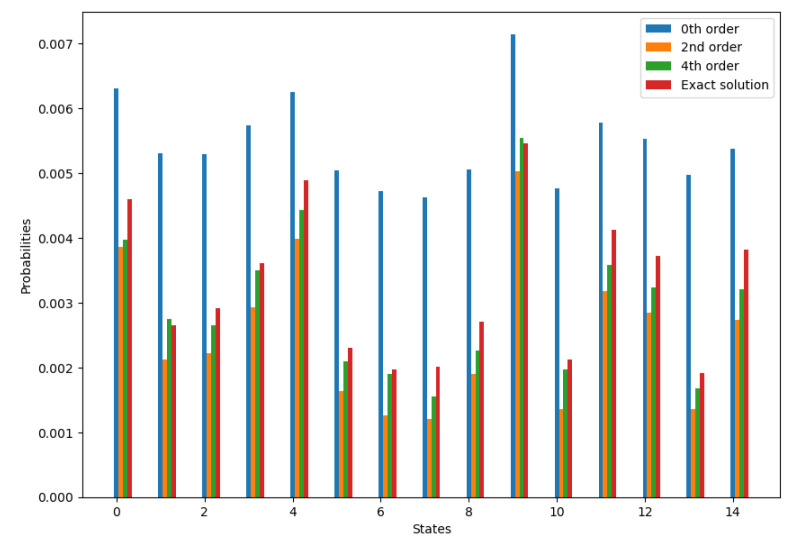
Probability calculation for 5 modes for different 2-photon output states.

**Figure 2:**
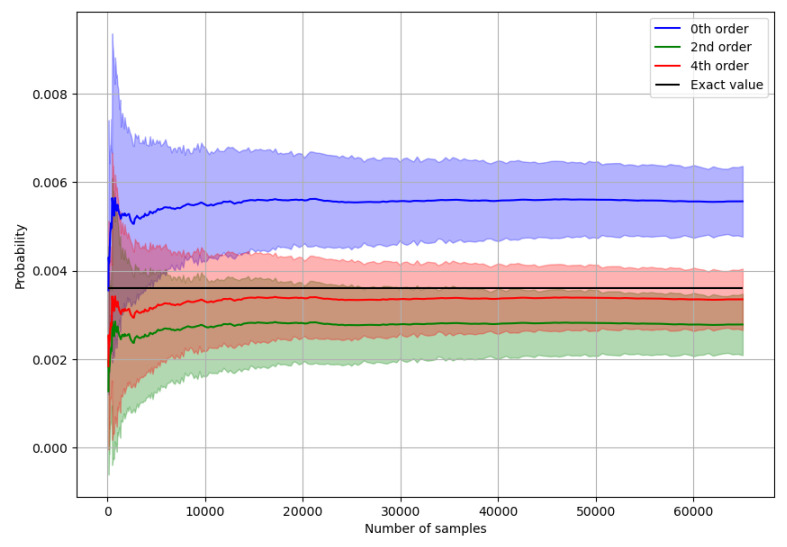
Graph of the average probability and the standard deviation calculated up to different orders for different numbers of samples. The state for this graph is 2-photon.

**Figure 3:**
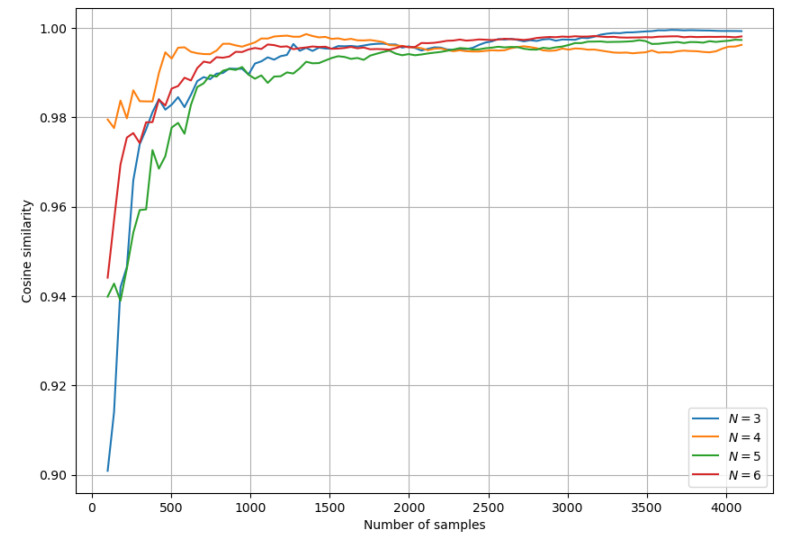
Convergence of the cosine similarity between estimated probability distribution over the set of all 2-photon states and ground truth for different *N*.

These results show that for calculating a single output state probability accurately, the number of samples needs to be on the order of $10^{4}$. Below are the results of using fewer samples per state, but instead of comparing individual probabilities, we look at the cosine similarity between the exact and approximated probability distributions over all two-photon states, Figure 1, Figure 2 and Figure 3.

**Figure 4:**
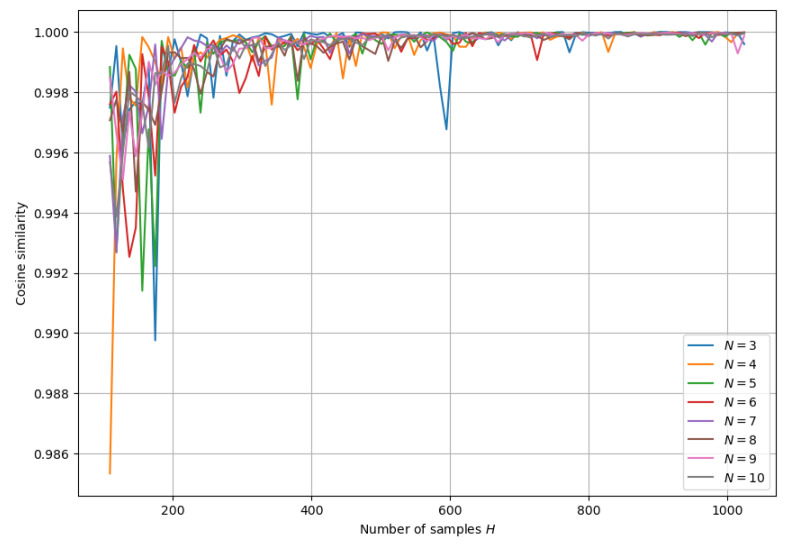
Cosine similarity between probability distribution over the set of all 2-photon states after *H* samples and after H+10 samples for different *N*.

The above graph suggests that the number of samples per state needed to approximate the distribution does not depend much on *N*. It is computationally hard to check this when comparing to the exact solution, but if we assume that the cosine similarity converges to a value close to 1, we can estimate how quickly it converges. Below, we look at the cosine similarity between a distribution calculated using *H* samples per state and a distribution calculated with H+ΔH samples per state for different *H*, where we choose ΔH=10. This allows us to estimate how much the distribution changes with ΔH new samples: if the cosine similarity is close to 1, then new samples do not alter the distribution significantly. Figure 4 suggests more strongly that the number of samples per state required for accurate approximation is not really influenced by *N*. This can be explained by the fact that the number of two-photon states increases with *N*. If the number of samples per state is constant, then the total number of samples increases with *N*.

**Figure 5:**
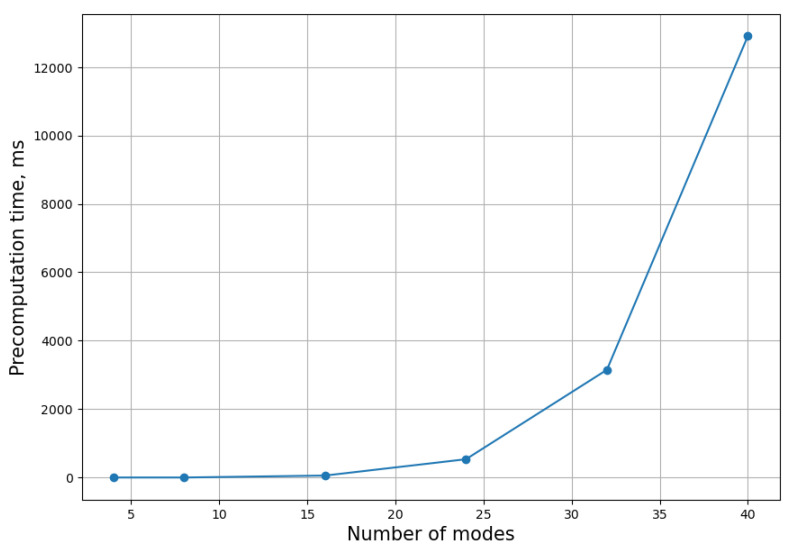
Precomputation time on an Intel i5 CPU in ms versus the number of modes.

**Figure 6:**
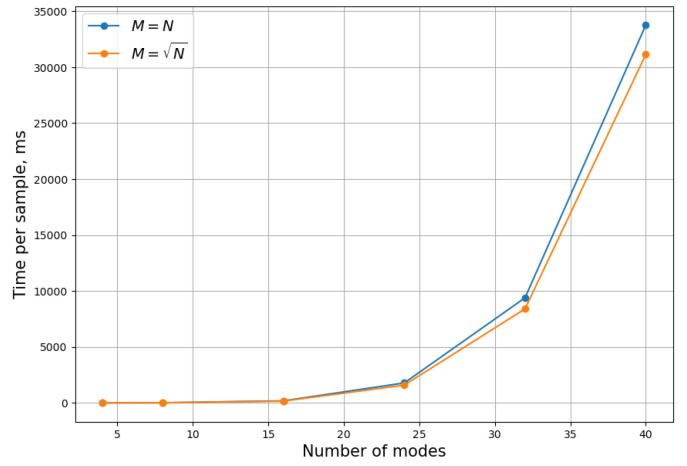
Average time per sample on an Intel i5 CPU versus the number of modes for states with different photon numbers.

Below are benchmark results that show the average precomputation time, which depends only on *N*, and the time per sample, which depends on *N* and the amount of photons *M* in the target state, Figure 5 and Figure 6.

These results show that even N=40 mode GBS can be simulated on an average laptop using this algorithm.

A direct comparison of the performance of our method with other published results is problematic, since our algorithm calculates the probability of output states rather than directly sampling states from some approximate distribution. The closest algorithm is described in [16]. However, the performance comparison is still problematic because the error bars of the two methods cannot be compared directly. Nevertheless, judging by Figure 9 from [16], our algorithm is more memory-efficient, since it uses about 0.1 GB of memory to run in conditions where the transmission rate is 0.5 and the number of modes is 40, whereas the algorithm [16] uses 1 TB, and the memory requirement grows exponentially with the number of modes.

## 9. Conclusions

In this paper, we have presented a new algorithm for the approximate calculation of the probability of observing a given output state in the Gaussian boson sampling instance. We have discussed various implementation details that help to reduce the number of operations needed to calculate each probability sample. We also analyze the total computational complexity both of the calculations that need to be carried out once for each specific problem and of computing each probability sample.

This algorithm relies on the Taylor series expansion where the “perturbation” parameter is dependent on the problem conditions. The algorithm consists of calculating the terms of this Taylor series up to some finite order. For a fixed maximum order, the computational complexity of the algorithm is polynomial in *N*.

We have demonstrated that increasing the maximum order does increase the accuracy of the answer. We have also measured the precomputation and sampling time for a regular CPU, showing that even large instances of Gaussian boson sampling (N≈40) can be solved in reasonable time.

We have considered recent GBS experiments and estimated the parameters of the problem for those conditions. We conclude that the contribution of the terms that are discarded when the calculation completed up to the second order is less than 5%, and if the calculation is completed up to the fourth order, this number drops to 1%.

## Data Availability

Data and program code are available upon reasonable request.
